# Healthy subjects with lax knees use less knee flexion rather than muscle control to limit anterior tibia translation during landing

**DOI:** 10.1186/s40634-020-00246-6

**Published:** 2020-05-15

**Authors:** Michèle N. J. Keizer, Juha M. Hijmans, Alli Gokeler, Anne Benjaminse, Egbert Otten

**Affiliations:** 1 Center for Human Movement Sciences, University of Groningen, University Medical Center Groningen, UMCG sector F, FA 23, PO Box 219, Groningen, 9713AV The Netherlands; 2 Department of Rehabilitation Medicine, University of Groningen, University Medical Center Groningen, Groningen, The Netherlands; 3 Luxembourg Institute of Research in Orthopedics, Sports Medicine and Science (LIROMS), Luxembourg, Luxembourg; 4 Department Exercise & Health, Exercise Science and Neuroscience, University of Paderborn, Paderborn, Germany; 5 School of Sport Studies, Hanze University Groningen, Groningen, The Netherlands

**Keywords:** Knee, Knee laxity, Muscle activity, Motor control

## Abstract

**Purpose:**

It has been reported that there is no correlation between anterior tibia translation (ATT) in passive and dynamic situations. Passive ATT (ATTp) may be different to dynamic ATT (ATTd) due to muscle activation patterns. This study aimed to investigate whether muscle activation during jumping can control ATT in healthy participants.

**Methods:**

ATTp of twenty-one healthy participants was measured using a KT-1000 arthrometer. All participants performed single leg hops for distance during which ATTd, knee flexion angles and knee flexion moments were measured using a 3D motion capture system. During both tests, sEMG signals were recorded.

**Results:**

A negative correlation was found between ATTp and the maximal ATTd (r = − 0.47, *p* = 0.028). An N-Way ANOVA showed that larger semitendinosus activity was seen when ATTd was larger, while less biceps femoris activity and rectus femoris activity were seen. Moreover, larger knee extension moment, knee flexion angle and ground reaction force in the anterior-posterior direction were seen when ATTd was larger.

**Conclusion:**

Participants with more ATTp showed smaller ATTd during jump landing. Muscle activation did not contribute to reduce ATTd during impact of a jump-landing at the observed knee angles. However, subjects with large ATTp landed with less knee flexion and consequently showed less ATTd. The results of this study give information on how healthy people control knee laxity during jump-landing.

**Level of evidence:**

III

## Background

Passive anterior tibia translation (ATTp) is often studied in literature, for example in people with hypermobility [1], anterior cruciate ligament (ACL) injured patients [2], or after a total knee arthroplasty [3]. ATTp, however, only gives information about knee laxity in situations where muscle activation and extern forces are absent or minimal. People with large ATTp may compensate for knee laxity by using effective muscle activation patterns in dynamic tasks or by amending their kinematics and kinetics in such a way that anterior tibia translation is limited. Therefore, anterior tibia translation in dynamic situations (ATTd) may give new information additional to ATTp. This can also be suggested by the absence of correlation between ATTp and ATTd found during normal gait, active extension, heel raises, cycling, one-legged squat and chair squat [4, 5]. This absence of correlation may be due to the contribution of muscle activation patterns and external forces in a dynamic situation. Previous studies found a relation between ATTp and pre-activation of the muscles [6–8], and ATTp and hamstrings activity [9]. Computer models showed that simulated hamstrings activity reduces the ATTd [10], and also showed that muscle activation patterns influence ATTd.

To the best of our knowledge, in literature no information is available on whether there is an in vivo correlation between ATTd and muscle activation patterns and between ATTd and knee kinetics in healthy people. Such information will enlarge the knowledge about how healthy people control knee laxity and may give us valuable information for people with hypermobility, with knee injuries or for ACL injury prevention programs. Those people, especially when large ATTp is observed, may be able to learn effective muscle activation patterns and landing strategies to limit ATTd. The present study will add to the current literature insight into the control of ATTd by muscle recruitment, kinematics and kinetics during a jumping task in healthy people. The aims of this study were to investigate:

1. Whether there is a correlation between ATTd and ATTp. To verify whether the absence of correlation between ATTd and ATTp found in literature holds during jump landing.
2. Whether quadriceps, hamstrings, and gastrocnemius activity are correlated with ATTd.
3. Whether the knee flexion angle and knee flexion moment are correlated with ATTd.

We hypothesized that quadriceps activity will increase ATTd, and hamstrings and gastrocnemius activity will decrease ATTd due to their anatomical insertions and lever arms. Moreover, we hypothesized that landing with more flexed knees and larger knee flexion moment will increase ATTd.

## Methods

A study was conducted at the motion lab of the UMCG department of rehabilitation medicine. The study design, procedure, and protocol are approved by the local Ethics Committee (ECB number: 2016.12.06.2 R2). All participants were informed about the procedures and the aim of the study by e-mail and signed an informed consent form.

### Participants

Twenty-one healthy participants (13 women and 8 men) who participated in recreational team sports (see Table 1) at least twice a week, and in addition played a match at least once a week, were included in the study. Moreover, the participants had to be between 18 and 45 years of age. Participants with any history of knee trauma, previous lower limb surgery, or self-reported disorders of the leg were excluded (Table 2).

**Table 1:** Sports of the participants

sport	n
Football	10
Volleyball	3
Korfball	2
Hockey	5
Handball	1
Total	21

**Table 2:** Baseline characteristics

	Mean +/− StD [range]
Age (years)	21 +/− 2.48 [18–26]
Mass (kg)	71.7 +/− 8.32 [60.7–91.3]
Height (mm)	178.3 +/− 2.37 [165–197.5]
BMI (kg/m^2^)	23.7 +/− 2.94 [19.1–32.8]
Hours of sport (a week)	5.9 +/− 2.37 [3–13]
Tested leg (right/left)	20/1

### Evaluation protocol

Each participant was measured in a single session. The passive test (condition 1) and the SLHD task (condition 2) were performed in a random order. The same researcher performed all procedures for every participant: electrode placements, marker placements and measurements.

First, sEMG-electrodes surface electromyographic (sEMG; Cometa Wave Plus Wireless sEMG system, Cisliano Milano, Italy) were attached according to SENIAM guidelines [11]. The skin was prepared by being shaved and cleaned with alcohol. All EMG-electrode pairs were placed along the length of the muscle fibers on the bulk of the muscles to reduce cross-talking [11]. For condition 1, the patterns of muscle activation were determined using the electrical signals of the medial hamstring (MH), lateral hamstring (LH), rectus femoris (RF), vastus medialis (VM), and vastus lateralis (VL) using sEMG. The patterns of muscle activation of the gastrocnemius medialis (GM) and gastrocnemius lateralis (GL) were not measured in condition 1 because of interference of the attachment of the KT-1000. For condition 2, the patterns of muscle activation were determined using the electrical signals of the MH, LH, RF, VM, VL, GM and GL. The sEMG signals were recorded at a sampling frequency of 1000 Hz.

During condition 1 (passive), ATTp was measured using a KT-1000 arthrometer (MEDmetric Corp, San Diego, California, USA) at a force of 133 N with the knee supported at approximately 30 degrees of flexion. The participants were laying supine and were instructed to relax their leg which the examiner verified by observing the sEMG recordings. This test was repeated three times and the average was taken.

**Fig. 1:**
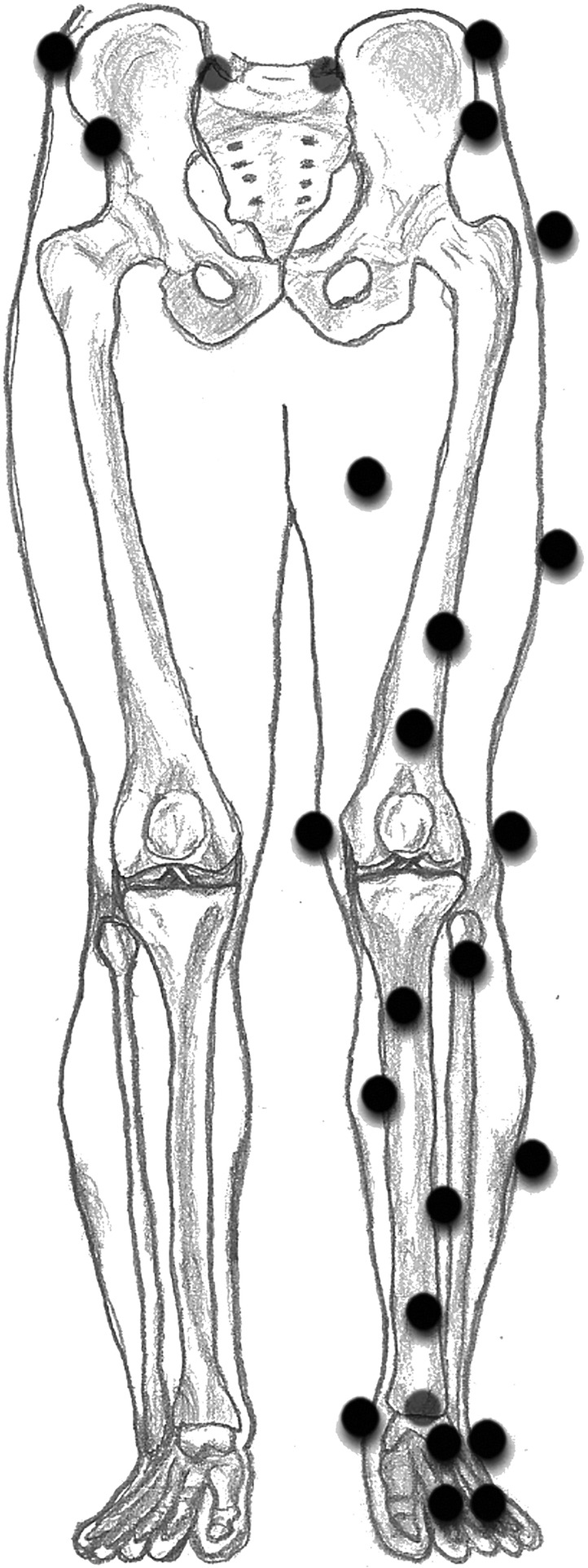
Marker placement. Markers were attached on the right and left anterior and posterior superior iliac spine, the right and left iliac crest, the greater trochanter, the medial and lateral epicondyles of the knee, the medial and lateral malleoli of the ankle, the heel, anterior of the talus bone and the first and fifth metatarsophalangeal joints. Besides, two additional markers were attached to the pelvis, two to the thigh, and six additional markers were attached to the shank (adapted from Boeth et al. (2013))

For condition 2 (dynamic), retroreflective markers were attached to the tested leg, the dominant leg of the participant (the leg that the participant prefers to use when kicking a ball [12]). Markers were attached as shown in Fig. 1 (adapted from Boeth et al. [13]). The 3D marker positions were measured with an 8-camera three-dimensional motion capture system (VICON MX_3+_; VICON Motion Systems Ltd., Oxford, UK) at a frequency of 100 Hz. After attaching markers, calibration frames of a flexion-extension movement and a star-arc movement, as prescribed by the manual of VICON, were performed to be able to identify the joint hip and knee centers and axes of rotation of the knee [14, 15]. Then, the participants performed SLHD wearing sports shoes and with their arms in free motion. First, three practice SLHD were performed. The participants started on their tested leg in a stationary posture and jumped as far as possible in a horizontal direction. The participants had to stand still on the same leg after landing for a minimum of three seconds. The distance of the furthest practice SLHD was used for the starting distance from the force plate. Next, ten successful SLHD were performed.

### Data analysis

The data were processed using a customized MATLAB (version 9.4, The MathWorks Inc., Natick, Massachusetts) script. The 3D marker position data were filtered using a convolution filter with low pass frequency of 10 Hz with zero lag, while gaps in the data of a maximum of 10 frames were filled with a quadratic spline interpolation. ATTd was determined based on a combination of the optimal common shape technique, symmetrical axis of rotation approach, and symmetrical center of rotation estimation combined [13]. For quantifications of ATTd and knee angles see Keizer and Otten [16]. It should be noted that results from this method should be taken with caution when transients are below 2.32 mm [16]. However, the intraclass correlation coefficient between observers who placed the markers is higher than 0.8 [17]. Knee flexion moment was calculated from the GRF vector and its lever arm to the center of the knee of the stance leg. ATTd, knee flexion angle, and knee flexion moment during each SLHD were determined for 1.5 s before the instant of first ground contact until 1.5 s after that instant. The time point of first ground contact was determined as the time where the vertical GRF on the force plate was at least 5 % of the body weight.

Muscle activity around the instant of first ground contact, taking into account an electromechanical delay of 50 ms [18], was rectified and filtered using a fourth order low pass frequency Butterworth filter at 6 Hz with zero lag. Muscle activity was scaled to a percentage of the mean muscle activity during the SLHD for each participant to reduce the influence of body fat.

### Statistical analysis

An a-priori power analysis based on the correlation between ATTp and ATTd of a healthy knees (contralateral knees of ACL injured patients; R^2^ = 0.34) [13], indicated that a total sample size of 18 participants would be required to achieve statistical significance at a 0.05 level with 80% power.

The data were analyzed using the Statistics Toolbox from MATLAB version 9.7 (The MathWorks Inc., Natick, Massachusetts). Pearson correlation analyses were performed between ATTp and maximal ATTd, and ATTp and range of ATTd.

In addition, an N-Way ANOVA was performed using a type II sum of squares and no interactions. For this analysis data from initial contact until 0.25 s after initial contact was used. The dependent variable was the ATTd and the independent variables were the activity of the independent muscles, the knee flexion angle, the knee extension moment and the ground reaction force rotated towards the tibia system in the medial-lateral and anterior-posterior direction. All variables were normalized to a scale of 0 to 1 by dividing their values by their maximal value during a session.

Correlations were considered to be significant with an alpha of ≤0.05. If a correlation was significant, a correlation coefficient of 0.2–0.49, 0.5–0.79 and 0.8–1 were considered to represent a weak, a moderate and a strong association, respectively [19].

## Results

### Passive and dynamic ATT

The mean ATTp was 3.4 mm (range: 0.9–8.8 mm). During the passive test, no more muscle activity than noise was found in a flat background signal of the sEMG.

**Fig. 2:**
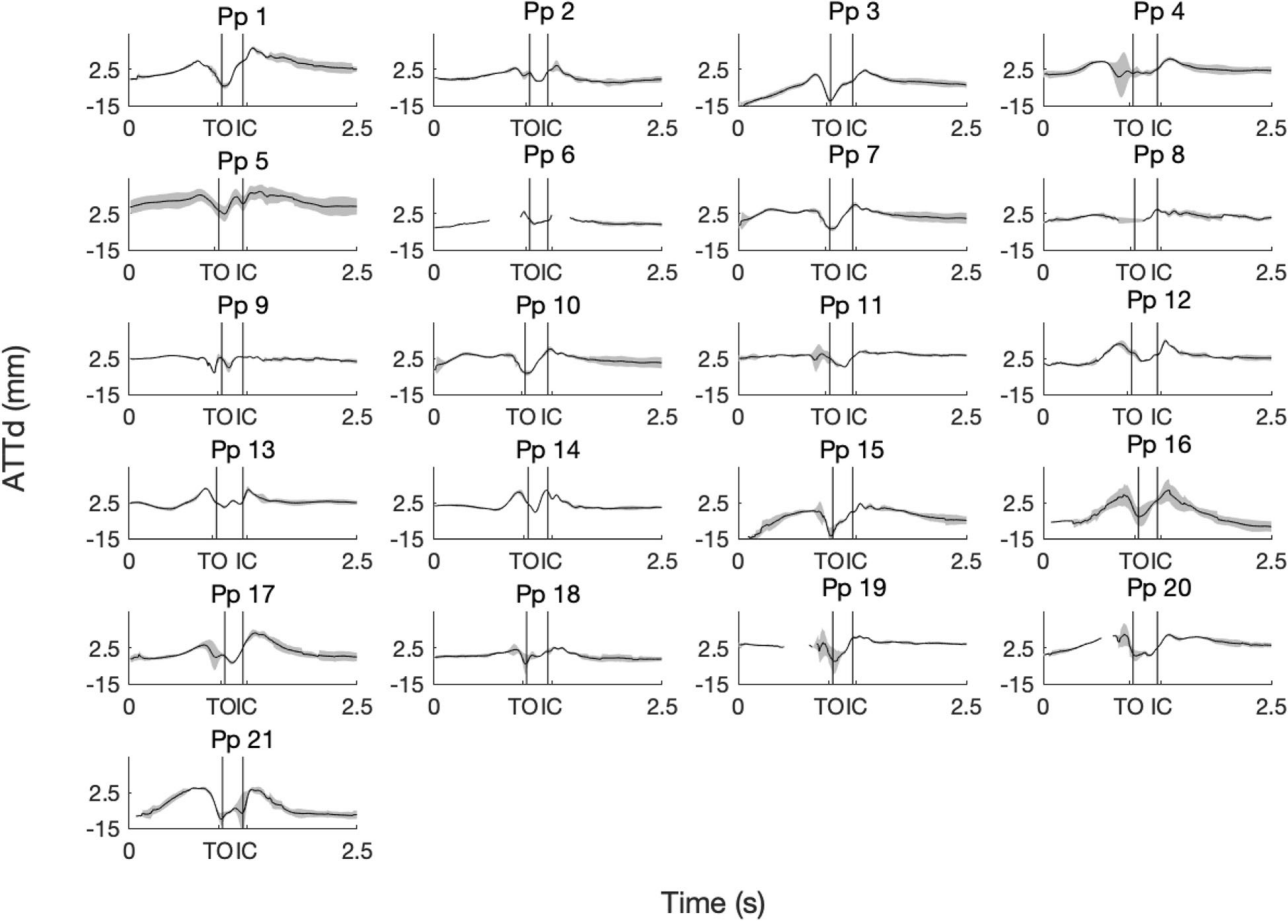
Mean and standard deviations of the dynamic anterior tibia translation (ATTd) of ten trials of a single hop for distance of all participants (Pp). TO: toe-off; IC: initial ground contact

**Fig. 3:**
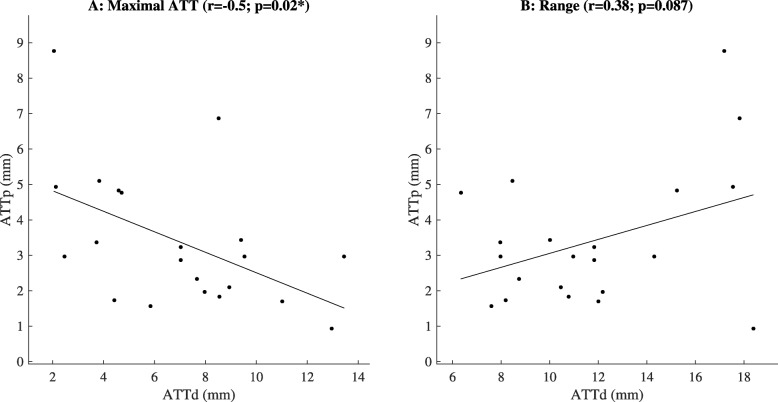
Passive anterior tibia translation (ATTp; KT-1000 arthrometer) v.s. the maximal dynamic anterior tibia translation (ATTd) and the range of dynamic anterior posterior tibia translation during a single hop for distance; *: significant

The ATTd for each participant is presented in Fig. 2. A weak negative correlation was found between ATTp and maximal ATTd (r = − 0.47, *p* = 0.028; Fig. 3a). No correlation was found between ATTp and the range of the anterior posterior tibia translation during jump landing (r = 0.38, *p* = 0.087; Fig. 3b).

### Control of ATT in a dynamic situation

In Table 3 the sum of squares, mean of squares, F-value, *p*-value and weight coefficients of the N-Way ANOVA are presented. The knee extension moment, knee flexion angle, GRFap, ST activity, BF activity and RF activity resulted in significant effects on ATTd.

**Table 3:** Sum of squares, degrees of freedom, mean squares, F-values, *p*-values and coefficient of the N-way ANOVA with the dependent variable being dynamic anterior tibia translation

Variable	Sum of squares (type II)	d.f.	Mean squares	F	p-value	Weight coefficient
KM	49.3	1	49.26	5.36	0.0208*	3.9112
KA	796.3	1	796.344	86.69	< 0.000*	8.0731
GRFml	4.1	1	4.056	0.44	N.S.	0.0004
GRFap	158.7	1	158.699	17.28	< 0.000*	5.8964
ST	241.5	1	241.537	26.3	< 0.000*	5.8438
BF	36.6	1	36.636	3.99	0.0461*	− 2.1236
GM	11.7	1	11.704	1.27	N.S.	1.3573
GL	5.7	1	5.657	0.62	N.S.	−1.6735
RF	83.7	1	83.734	9.12	0.0026*	− 3.148
VM	6.8	1	6.796	0.71	N.S.	1.4945
VL	2.7	1	2.699	0.29	N.S.	−1.0857
Error	9507.1	1035	9.186			
Total	14,429.5	1046				

*:significant; *KM* knee extension moment, *KA* knee flexion angle, *GRFml* ground reaction force in the medial-lateral direction, *GRFap* ground reaction force in the anterior-posterior direction, *ST* semitendinosus, *BF* biceps femoris, *GM* gastrocnemius medialis, *GL* gastrocnemius lateralis, *RF* rectus femoris, *VM* vastus medialis, *VL* vastus lateralis

## Discussion

The most important findings of this study were:

1. A negative correlation between ATTp and maximal ATTd.
2. That larger ST activity was seen when ATTd was higher, while BF activity and RF activity were lower.
3. That higher knee extension moment, knee flexion angle and GRFap were seen when ATTd was higher.

### ATT compared to literature

A review showed a range of ATTp of approximately 2.5–8.4 mm in healthy knees [20]. The present study found a range of 0.9–8.8 mm, which is comparable to the literature study. A lack of golden standard of ATTd measurement makes it difficult to verify the outcomes of the methods developed by Boeth et al. [13]. However, the ATTd found in our study is comparable with that of previous studies. In our study the mean range of ATTd was 11.5 mm (− 4.7 to 6.8 mm). Previous studies found an absolute range of ATTd using bi-planar fluoroscopy model based data during running of around 10 mm (8 to 18 mm) [21] and +/− 25 mm [22], and using the same methods as in the present study around 12 mm (− 2 to 10 mm) [16]; all in healthy subjects.

### Correlation between ATTd and ATTp

The present study revealed a significant negative correlation between ATTp and maximal ATTd. In contrast with these findings, Boeth et al. [13] did find a significant positive correlation between the ATTp measured also using the KT-1000 arthrometer and the range of the anterior posterior tibia translation during walking. This difference in results may be related to the task: jumping is more challenging in terms of net joint moments of force and anterior tibia shear force, which may allow less room for phasic co-activation of the muscles in a much shorter time window in which the joint load is growing. In addition, others did not find a correlation between ATTp and ATTd (during gait) measured using a CA-4000 electrogoniometer in ACL deficient knees [5]. This may be due to differences in measurement method, due to the task or due to the injury. During walking ATTd may not be maximal as the impact on the knee is small and a knee injury may result in an inhomogeneous group of participants.

The finding of the current study that people with high ATTp tent to show low ATTd suggest that passive ATT tests are not representative for ATTd, and that people with high ATTp may be able to control their knee laxity during jump landing, i.e. by using adequate muscle activation patterns or kinematics.

### Active control of ATTd

Surprisingly, the effect of the knee flexion angle on the ATTd and the effect of extension moment on the ATTd were higher than the effect of muscle activation on ATTd. This result might imply that muscle activation patterns do not contribute to reduce ATTd in healthy people during a SLHD landing. This can also be seen in the sign of the predictive weight coefficient of the ST and RF activity with ATTd. ST activity has a positive predictive weight coefficient whereas RF activity has a negative predictive weight coefficient on ATTd, which is in line with the fact that the hamstrings are known to pull the tibia posteriorly relative to the femur and the quadriceps pull the tibia anteriorly [23, 24]. However, according to measurements of Kirkendall and Garrett [25] landing with low knee flexion does increase the knee extensor activity and landing with higher knee flexion does increase the hamstring activity. This is in line with our results. These results might mean that the pattern of muscle activity at the observed net knee moment is unable to limit the ATTd at that knee angle. Participants with a large ATTp use less knee flexion while landing and have less ATTd.

In literature it is shown that ACL strain increases when the knee is more extended (between 0 and 30 degrees) in cadaveric knees using a strain transducer on the anteromedial bundle of the ACL [26, 27] and in healthy knees using an MRI and fluoroscopy based model during jump landing [28]. Therefore, it is previously suggested that landing with a more flexed knee (so called soft landing) may protect the ACL since it is not strained [29–31]. In physiotherapy after an ACL injury and reconstruction as well as in ACL injury prevention, people are therefore instructed to land with more knee flexion to protect the ACL [32, 33]. The predictive weight coefficient between knee flexion angle and ATTd was positive. This might imply that there is more room for ATTd during jump landing when the knee is more flexed. When there is more room for ATTd the possible anterior tibia acceleration might be higher and therefore the sudden impact of the tibia on the ACL strain might be higher during uncontrolled movements. For example, in expert skiers it is shown that the ACL can be torn when the quadriceps contract in a short time period while the knee is in a high flexion which results in a high anterior tibia acceleration [34]. Note that this all depends on the inertial properties of the elements and their accelerations. Nevertheless, a numbers of studies suggest that most ACL injuries occur while the knee is near full extension or in hyperextension [35, 36].

### Future research and limitations

Further research is necessary to corroborate or reject our findings that landing kinematics and kinetics are more important in the control of ATTd than muscle activation. Perhaps in people with larger knee laxity, a suitable landing strategy is already found autonomously. Also, future studies could investigate if patients after an ACL injury can compensate for the dynamic knee laxity using effective landing kinematics, kinetics and muscle activation patterns. Such studies can be designed to investigate if patients who can cope with the injury may compensate for the available passive knee laxity by using effective landing strategies and muscle activation patterns in a dynamic situation whereas patients who cannot cope with the injury might not be able to compensate for the results of the injury. Also, more research is needed on the contribution of limiting ATT by respectively strain in the ACL and muscle forces. This requires a good 3D model fed by material properties, geometrical data and experimental data in dynamical situations.

Other factors such as anatomical differences, i.e. the slope of the tibia plateau, might also be important for the observed ATTd. Shao et al. [37] reported by using a biomechanical computer model that ATT is influenced by the slope of the tibia plateau. Further research is necessary to investigate the influence of anatomical differences on the ATTd.

Some limitations of this study need to be addressed. There may be errors in the results of ATTd due to wobbling masses of the muscles in the upper and lower leg on which the optical markers were affixed, falsely represented as ATTd. However, a sensitivity analysis of the methods used in the present study revealed that only transients less than 2.32 mm should be taken with caution [16]. A second limitation is the method of normalization of muscle activity. We have chosen to normalize the muscle activity to the percentage of the mean muscle activity during the SLHD. This normalized muscle activity might be more comparable between participants than the absolute muscle activity since the influence of variables like conductance and body fat are canceled. We have chosen not to normalize to a maximal voluntary contraction task, as we found that some participants showed different isometric activation strategies than others in those tasks. A third limitation might be the sample size. Even though we met the number of participants calculated with a power calculation, the variety in the ATTd within the study group was high. This might explain the lack of correlations or when significant, only weak or moderate correlations.

## Conclusion

The results of this study show that participants who have more knee laxity during the passive test have smaller ATTd during the SLHD. Subjects with a large ATTp land with less knee flexion and have less ATTd. Participants did not use muscle activation at impact in such a way that ATTd is reduced during a jump-landing task. The pattern of muscle activity at the observed knee moment is unable to limit the ATTd at that knee angle.

## Data Availability

The datasets used and/or analysed during the current study are available from the corresponding author on reasonable request.

## Abbreviations

ATTp: Passive anterior tibia translation
ATTd: Dynamic anterior tibia translation
ACL: Anterior cruciate ligament
SLHD: Single leg hop for distance
MH: Medial hamstring
LH: Lateral hamstring
RF: Rectus femoris
VM: Vastus medialis
VL: Vastus lateralis
GM: Gastrocnemius medialis
GL: Gastrocnemius lateralis
GRFap: anterior-Posterior component of the ground reaction forces
GRFml: Medial-lateral component of the ground reaction forces
sEMG: Surface electromyographic
